# Mapping mHealth Research: A Decade of Evolution

**DOI:** 10.2196/jmir.2430

**Published:** 2013-05-21

**Authors:** Maddalena Fiordelli, Nicola Diviani, Peter J Schulz

**Affiliations:** ^1^Institute of Communication and HealthFaculty of Communication SciencesUniversity of LuganoLuganoSwitzerland

**Keywords:** mHealth, systematic review, health outcomes

## Abstract

**Background:**

For the last decade, mHealth has constantly expanded as a part of eHealth. Mobile applications for health have the potential to target heterogeneous audiences and address specific needs in different situations, with diverse outcomes, and to complement highly developed health care technologies. The market is rapidly evolving, making countless new mobile technologies potentially available to the health care system; however, systematic research on the impact of these technologies on health outcomes remains scarce.

**Objective:**

To provide a comprehensive view of the field of mHealth research to date and to understand whether and how the new generation of smartphones has triggered research, since their introduction 5 years ago. Specifically, we focused on studies aiming to evaluate the impact of mobile phones on health, and we sought to identify the main areas of health care delivery where mobile technologies can have an impact.

**Methods:**

A systematic literature review was conducted on the impact of mobile phones and smartphones in health care. Abstracts and articles were categorized using typologies that were partly adapted from existing literature and partly created inductively from publications included in the review.

**Results:**

The final sample consisted of 117 articles published between 2002 and 2012. The majority of them were published in the second half of our observation period, with a clear upsurge between 2007 and 2008, when the number of articles almost doubled. The articles were published in 77 different journals, mostly from the field of medicine or technology and medicine. Although the range of health conditions addressed was very wide, a clear focus on chronic conditions was noted. The research methodology of these studies was mostly clinical trials and pilot studies, but new designs were introduced in the second half of our observation period. The size of the samples drawn to test mobile health applications also increased over time. The majority of the studies tested basic mobile phone features (eg, text messaging), while only a few assessed the impact of smartphone apps. Regarding the investigated outcomes, we observed a shift from assessment of the technology itself to assessment of its impact. The outcome measures used in the studies were mostly clinical, including both self-reported and objective measures.

**Conclusions:**

Research interest in mHealth is growing, together with an increasing complexity in research designs and aim specifications, as well as a diversification of the impact areas. However, new opportunities offered by new mobile technologies do not seem to have been explored thus far. Mapping the evolution of the field allows a better understanding of its strengths and weaknesses and can inform future developments.

## Introduction

In the last decade, mobile health (mHealth), the branch of eHealth broadly defined as “the use of mobile computing and communication technologies in health care and public health” [1], has been constantly expanding. Mobile applications for health can target heterogeneous audiences such as doctors, nurses, patients, or even healthy people [1]. Different features of mobile phones may address specific needs in different situations. Available literature suggests that the use of mobile phones serves a wide variety of purposes [2], such as smoking cessation, weight loss, diet and physical activity, treatment adherence, and disease management. The biggest advantages of using mobile devices, and in particular mobile phones, for health are that these devices are personal, intelligent, connected, and always with people [3,4]. Therefore, they can serve patients both in everyday life and during hospitalization or rehabilitation, as well as health care providers during emergency or routine visits. Current evidence suggests that the use of mobile technology can improve diagnosis and compliance with treatment guidelines, as well as patient information, and can increase administrative efficiency [5]. In particular, short message service (SMS) text messaging reminders have been shown to be a simple and efficient option for health services to use in order to improve service delivery, resulting in health benefits for the patients who receive them [6]. Mobile phone technologies have also been shown to be effective in smoking cessation, weight loss, physical activity, diabetes management, STD prevention and treatment, and hypertension [7].

The mobile phone market is constantly evolving. The first digital mobile phones appeared in the early 1990s, and since then, mobile technology has continued to be refined thanks to the development of new features and better networks. Current smartphones have been defined as “mobile telephones with computer features that may enable them to interact with computerized systems, send e-mails, and access the web” [8]. Over a third of US mobile phone users own a smartphone [3,9], and it is estimated that 67.6% of adults worldwide own a mobile phone [2,10], making it the most equitable communication technology [1]. It has been argued that mobile phones could be a solution to overcome the traditional digital divide derived from the introduction of the Internet because they provide new opportunities to reach underserved and previously unreachable parts of the population worldwide, especially in developing countries [2].

Mobile technology, with its diffusion and characteristics, holds a great potential for health care applications. However the use of mobile phones in health care delivery has not been fully explored, and the diverse outcomes of mHealth have barely been documented. Although some literature reviews cover one part or the other of the field [6,11,12], an overall picture is still missing, possibly due to the field’s constant evolution. A recent methodological review sought to map the domain of mobile phone health interventions [13], but it relied on describing the design of the interventions, with a clear focus on technology, rather than the outcomes. As the authors stated, their motivation lay in the fact that “effectiveness reviews can be best done at the level of a particular pathology”, while they wanted to draw a more comprehensive taxonomy of the field.

The main objective of this paper, as stated in the title, is to map the field, but without omitting the outcome measures. This means that our intention is to investigate how the impact of mobile phones on health has been assessed in peer-reviewed scientific literature. In particular, we are interested in understanding the evolution over the past decade, how the interventions have been developed, the main health care delivery areas where the impact of mobile technologies has been assessed, the methodology and features used, and finally, the type of outcome measures and general impact of the intervention.

The second objective of this review is to understand, after the 5 years since the introduction of the new generation of smartphones (eg, the iPhone in 2007), whether and how these devices have triggered research. The appeal of these new devices resides in the fact that they include several computer-like built-in features (eg, the GPS or the accelerometer) allowing the monitoring of a whole series of behaviors. Additionally, new mobile operating systems allow users to customize their devices according to their needs, by downloading apps available for free or for a low price from a central store. Klasnja and Pratt named this kind of feature “native application” [13], which is a typical complex and sophisticated application that can be implemented on major smartphone platforms (iOS, Android, Symbian, BlackBerry, webOS, and Windows Phone). In 2012, smartphone users spent US $8 billion for paid apps in the top 5 app platforms, and the European mobile app market size reached €1.68 billion [14]. Therefore, iPhones and similar devices are potentially very interesting for application in health care—they already integrate most of the features that researchers previously had to add to traditional mobile phones in order to use them for health-related purposes and monitoring [15,16].

## Methods

The objective of this study was to provide a comprehensive picture of how the impact of mHealth was assessed in the scientific literature in its first decade of existence. For this purpose, a systematic literature review was conducted in which relevant studies were categorized in a two-step process. The first step included the review of the titles and abstracts of all publications that were identified as potentially relevant, with the goal of assessing whether they might meet the inclusion criteria for the systematic review. Selected abstracts were categorized at this stage using general typologies partly adapted from existing literature [1,2,12] and partly created inductively from a subsample of the publications. Categories referred to the type of methodology used, the impact area (ie, remote monitoring, data gathering, communication, self-management, training/education, improve adherence, health promotion), and the type of study. In a second step, all the publications not excluded during the abstract and title review stage underwent a full-text review. All publications that met all eligibility criteria (see below) made up the final sample.

### Search Strategy

In February 2012, five electronic databases (CINAHL, Communication and Mass Media Complete, PubMed, PsycINFO, and Web of Science) were systematically searched. The choice of databases was deemed to reflect the multidisciplinary nature of the field. Among the most used medical databases, we decided to include PubMed only, since it comprises MEDLINE, while Embase was excluded because it has a stronger drug coverage, which was not relevant for the purposes of our research. A list of keywords was created around the two domains of “health” and “mobile technology”. A search string was constructed using both the conjunction “AND” and the disjunction “OR” logical operators ([health OR medicine OR medical OR telemedicine OR health care OR “mHealth” OR “mobile health” OR “m‐health” OR “mobile‐health”] AND [“mobile phone” OR “cell phone” OR “cellphone” OR “cell‐phone” OR “smartphone” OR “iPhone” OR “blackberry” OR “android”]). The search was based on metadata, ie, title, abstract, and keywords. Reference lists of selected studies were also checked for other potentially relevant studies.

### Selection Criteria

Eligibility criteria for inclusion were as follows: records had to be written in English and discuss/acknowledge the role of mobile technology as a tool for promoting, managing, or monitoring health. This could include interventions, cross‐sectional studies, literature reviews, conceptual papers, etc. All articles dealing with health effects of mobile phones (eg, effects of non-ionizing radiation on health or effect of mobile phone use on adolescents) were excluded. Records had to be officially published, either online or in print in a peer-reviewed publication (ie, journal articles, book chapters, and published proceedings papers). This means that poster presentations, (extended) abstracts, and encyclopedia entries were excluded. No time restriction was given; all publication dates were eligible for inclusion. Also, there was no restriction on the field of studies, ie, records that could be classified as social sciences, humanities, medicine, and others were all included.

The exclusion criteria that accounted for the biggest number of excluded articles included the following: the study provided descriptive summaries of mHealth programs but failed to provide an evaluation of the program; study provided a short description of multiple mHealth programs without providing specific details on an mHealth intervention; and the study focused on mHealth application design. The title and abstract review allowed us to exclude system design articles and to better identify all the studies that involved people in the testing of the intervention. A full-text article review was therefore conducted only on studies evaluating and assessing mHealth applications. The categories for full-text review were the following: continent where the study took place, condition addressed, type of technology, features used, basis for the intervention development, study design, sample size, aim of the evaluation, outcome measures, and overall impact assessed.

## Results

**Figure 1:**
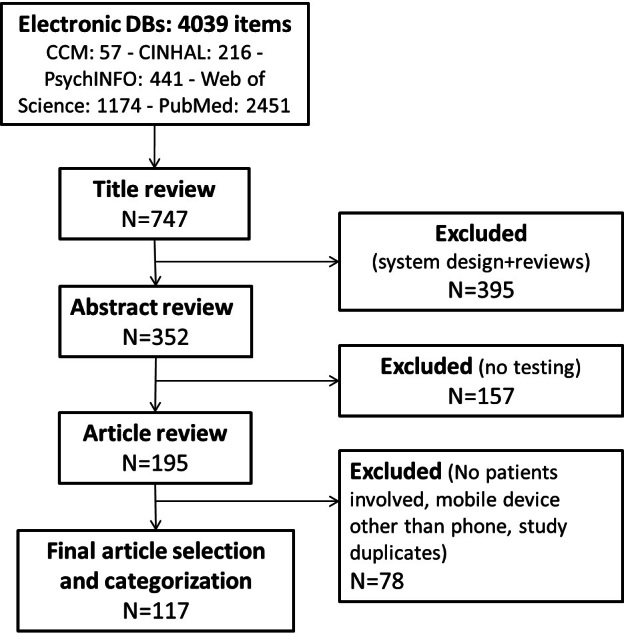
Summary of literature search and review process.

The flowchart in Figure 1 summarizes the different steps of the literature search and review process. A first search identified 4039 articles. After checking for duplicates, 747 articles met the predefined inclusion criteria. Initially, articles were categorized by type of study: quantitative, qualitative, mixed methods, review, and system design. Since articles in the last typology described the development of a mobile technology but did not include any actual testing, they were excluded from further analysis together with reviews, reducing the final sample to 352 articles. More than half of the 352 studies (56%) included at least some testing of a mobile phone application via proper interventions or in small samples. Most of the studies analyzed (86%) applied a quantitative methodology and were designed to address simultaneously one or more impact areas. An upsurge was noted, starting from 2008, when the articles doubled in comparison with the previous year, and this upward trend reached its maximum in 2011 (36% of the total in a 10-year time period). The search of scientific databases without a time limit yielded an article distribution on the topic over 10 years, from 2002 to 2012.

**Figure 2:**
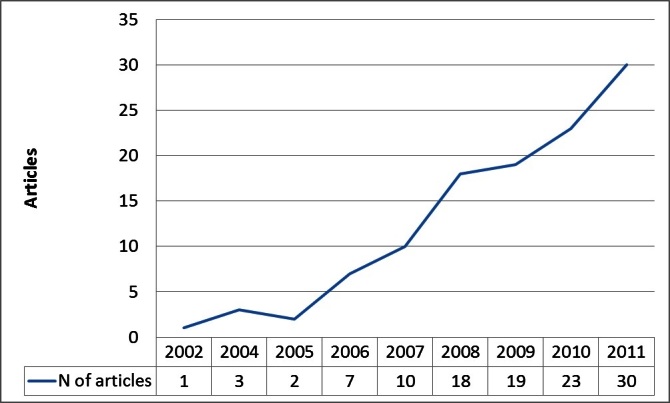
Distribution of articles over time.

The final sample for the full-text review included 117 [17-133] articles out of the 352 described above. After title and abstract review, an additional 157 articles were excluded because of no actual testing, while another 78 were excluded during full-text revision for different reasons (eg, no patients involved, mobile device other than phone, study duplicates). Looking at this past decade (Figure 2), we again observed an upsurge in the field: from 1 article in 2002 to 30 articles in 2011. The largest upsurge again came between 2007 and 2008, when the articles almost doubled, similar to what was already noted during the abstract review phase.

In order to better reflect the objectives of our review and to mirror the development over time, all the results are presented by splitting our observation period in two halves (2002-2007 and 2008-2012). The first period includes 23 articles, while the second period includes 94. The 117 articles in the final sample were published in 77 different journals, which can be grouped in four disciplinary fields: technology, medicine, social sciences, and the intersection between technology and medicine. During the first half of the observed decade, most of the articles on mHealth were published in medical journals (52%) and in journals focused on medicine and technology (44%).The remaining 4% of articles were published in journals focused only on technology. In the second half, the share of articles published in medical journals grew from 52% to 60%, while coverage of the topic by technology journals did not change (4%). At the same time, a decrease in the share of articles published by journals dealing with medicine and technology was observed (from 44% to 35%). In the second period, we found one article from a new disciplinary field, the social sciences.

The geographical areas focusing on this type of research were mainly Europe (34%) and North America (33%). However, if we look at results split by time periods, Europe’s interest seems to decrease from the first period (52%) to the second (30%), and the same happens in Asia (from 17% to 10%). A different picture can be found on all the other continents, where the number of studies in the field increased. This is the case in North America (from 17% to 37%), Australia (from 13% to 15%), and especially Africa (from 0% to 6%) and South America (from 0% to 2%).

**Figure 3:**
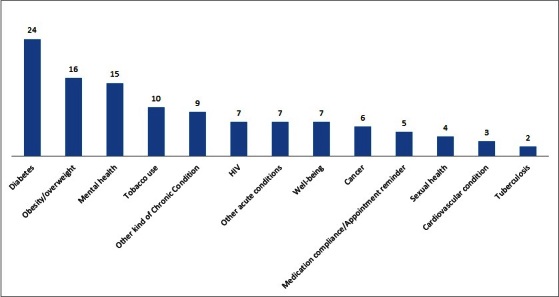
Conditions addressed by mHealth applications in mHealth research.

Specific health conditions addressed in the studies ranged from diabetes to mental health, from obesity to well-being and postoperative care. Figure 3 shows the number of articles for every health condition for which mHealth applications were studied. As shown in the graph, diabetes has received a great deal of attention. Moreover, after grouping the conditions into larger classes, it becomes clear that the focus of mHealth research is chronic conditions (74 studies), followed by prevention/well-being (22 studies), and acute conditions (21 studies).

In reviewing the background of the studies, we found that a description of the development of the intervention, and especially of how this was grounded, occurred more often in the second period (84%) than in the first (65%). During the last 5 years, only 1 study was uniquely theory-based (1%), while the majority was evidence-based (73%) or based both on theory and evidence (10%); 15 studies (16%) provided a more general description that was based on neither theory nor previous evidence.

From a methodological point of view, the majority of articles were clinical trials (50%), followed by pilot studies (44%). However, both of these study designs diminished over the last years of our observation period as new types of research designs were introduced, namely observational studies (2% of all articles in the second part of the observation period), case studies (2%), case series (2%), and cross-sectional studies (2%). The samples used to test mobile health applications were mostly small (less than 50 people) in both the first (61%) and the second half (49%) of our observation. Interestingly in the second half, the number of medium-sized samples increased (from 17% to 33%). Larger samples were used in 21 (19%) articles; however, they were more frequent in the first half of the observation (22%) than in the second half (18%).

Moving from research methodology to the actual target of investigation, ie, mobile phones, our classification highlighted a more rigorous and diversified description of the technology used in interventions. In recent years, new kinds of mobile phones have been used, such as smartphones (8%) and ad hoc phones (3%), which are devices developed specifically by the researchers to manage a specific condition. Unfortunately, the kind of mobile phone used was not even specified most of the time (71% of the overall sample).

We identified seven main categories of mobile features used in the studies, and an article could fall in one or more of these (ie, the categories were not mutually exclusive). Half of the studies (49%) applied text messaging, and 32% applied some features developed ad hoc for a specific condition. Add-ons (eg, a glucometer to measure blood sugar or a pedometer for physical activity) were used in 12% of the cases together with ad hoc features. Other features such as voice (10%), video (6%), and multimedia messaging service (MMS) (3%) were used less frequently. Native applications for smartphones were applied in 7 studies (6%) out of the 8 using smartphones. However, none of them applied already existing and publicly available apps.

The impact areas to which interventions were directed were coded into seven categories, again not mutually exclusive. The majority of articles addressed health promotion (38%) and self-management (33%), but also communication (22%), remote monitoring (21%), data gathering (21%), improvement of adherence (20%), and training/education (13%). The focus on most of these areas increased over time, eg, on self-management (from 30% to 33%) and communication (from 17% to 23%). Only health promotion (from 29% to 27%) and training/education (from 13% to 10%) had a slight decrease.

Regarding the aims of the interventions, both the evaluation of the technology itself (35%) and of its impact on health outcomes (43%) dominated in the first 5 years. In the second half, however, interest clearly moved toward evaluating the impact of mobile technology on health outcomes (73%). While the majority of the studies investigated only the impact of the mobile application on health outcomes (51%), some also assessed both the technology and its impact on health outcomes (22%).

Another point of interest was the outcome measure used to assess the impact of mobile phones. In the majority of cases, the outcome measures were a combination of both self-reported and objective data (44% of the overall sample). If we look at the evolution over time, self-reported measures increased (from 9% to 20%), whereas objective measures decreased slightly (from 39% to 36%), and this was also the case for the combination of self-reported and objective measures (from 52% to 43%).

Our examination of the type of data collected showed that clinical measures were often the only outcomes observed (30%), and this phenomenon increased over time (from 22% to 31%). 14% of the articles were focused only on user assessment of the technology, even if this decreased during the observation period (from 17% to 13%). Psychosocial measures were the outcome in 9% of the studies, and this increased slightly over time (from 9% to 10%). The remaining articles (53%) considered outcomes deriving from all possible combinations of these main three. The most frequent combination was clinical measures together with user assessment of technology (17%).

An overall positive impact of the intervention was described by a total of 69 studies (60%). In the first period, the impact of the interventions was mainly either mixed (43%) or positive (57%). In the second part of the observation, the number of interventions with a positive impact slightly increased (60%), while the number of those with mixed impact decreased (33%). In this second period, interventions with negative (6%) or no impact (1%) were reported as well.

## Discussion

This systematic literature review has found encouraging trends pointing toward the development of mHealth as an autonomous field of study, which is of interest to different disciplines. In the case of social sciences for instance, this is shown by the publication of a special issue of the *Journal of Health Communication* dedicated entirely to mHealth in May 2012. We took this into account but did not include it in the current review because of the date of publication.

The increase in the number of articles over the past decade indicates an increasing interest in peer-reviewed scientific literature on the topic. In particular, the number of articles almost doubled from 2007 to 2008. All continents engaged in research on mHealth during the last 5 years. Many different conditions were addressed by the studies analyzed, with an evident focus on chronic conditions. More recently, the development of the interventions became more accurately described and grounded on past evidence or theory. In the last decade, the first in the history of mHealth, new methodologies were applied to the field, and while the majority of the studies were pilot studies or clinical trials, some new approaches were introduced. At the same time, the samples used in such studies grew, thus pointing toward a more reliable assessment. Newer and more advanced technologies were tested; however, the potential of smartphones does not seem to have been fully exploited yet. Indeed, half of the included studies applied very basic features of mobile phones, such as text messaging, which corroborates evidence already established in the field [7]. One third of the studies used features from add-ons, and only a few other examples used video, images, or MMS. Mobile technology interventions were directed to different impact areas, and most frequently to health promotion and self-management. Interestingly, research moved from an evaluation of the technology itself to an assessment of its impact on health outcomes, and an increase in the number of interventions with an overall positive impact was observed. These observations point toward a field that is becoming more structured, coherent, and solid. Among the outcome measures used, both self-reported and objective measures could be found, and in some cases were combined. In the last 5 years, the use of self-reported measures increased. Clinical measures only were observed as outcomes in almost one third of the studies, and an increasing trend was evident. Consistent with the observed trend towards an assessment of the impact of technology, we noticed an increase in psychosocial and clinical measures, sometimes in combination with users’ assessment of the technology, and a decrease of assessments of the technology alone.

### Recommendations

Although these findings are encouraging and can be seen as indicators of a promising field, they highlight certain gaps that future research should address. So far, the focus of mHealth interventions has been on chronic conditions, similar to eHealth [134]. However, it would be advisable to explore the impact of mobile health for acute conditions as well. Because of their wireless cellular communication capability, mobile phones allow users to have continuous, interactive communication from any location. In our view, this characteristic of mobile phones makes them an ideal tool to address in real-time the specific needs of patients experiencing acute conditions.

Another recommendation is to address methodological issues such as whether clinical trials are the most suitable design to use at every stage of research in this field. The studies we analyzed showed a diversification toward the second half of the decade, which seems to reflect more positive outcomes and a stronger evidence-based development of the interventions. Consistent with the conclusions of other authors [3,5,7,135], we believe the field will greatly profit from a diversification in research methodologies and that the multidisciplinary approach offered by different areas could be a fertile ground for the development of the field, both from a theoretical and methodological perspective.

Our last recommendation is for research to fully exploit the potential of technologies, especially of smartphones. We expected in our review to find more results detailing applications of new built-in features, which are the “specialty” of smartphones. However we found that only a few interventions aimed at assessing the impact of native applications for smartphones had been reported so far in the literature. Moreover, in all the cases, the apps were not available to the public but had been created ad hoc for research purposes**.**

This last recommendation brings up a new topic of discussion. Currently, there are more than 15,000 health-related apps (free and paid) on app stores, but we were not able to find any study assessing any of them. So what is publicly available has not been evaluated, and what has been evaluated is not publicly available. At least three possible scenarios could explain the lack of scholarly interest in studying the effects of interventions based on publicly available apps. First, no one, so far, has conducted such studies. If this is true, it provides great opportunities for investigation in this area, since some basic features of mobile health such as text messaging have already proved to be effective. A second scenario could be that it is still too early for results of such assessments to have been published. However, this seems to be less plausible, because iPhones and similar devices have already been on the market for 5 years. Finally, it could be that studies evaluating the impact of native applications have indeed been published, but not in the peer-reviewed scientific literature. A search of the gray literature, such as more consumer-oriented magazines, websites, or blogs (eg, iMedicalApps) could then yield some results. If this last, and more plausible, explanation is true, then we need to document the exploitation of smartphones in a way that is easily accessible to the scientific community. If only a minority of good quality applications and solutions seek clinical mHealth research to prove effectiveness, we should question why this is happening. It is thus essential to understand why scientific literature is not keeping up with advancements of the field, to find where any discussion is taking place, and to find the evidence of apps’ effectiveness, which seems to be missing [3].

### Limitations

A limitation of our study, which is common to most systematic literature reviews, resides in the fact that the last articles analyzed were published in February 2012. In this constantly evolving field, this could make a difference, especially because we are well aware that the publication process is often lengthy and time-consuming, and therefore we could have missed some studies on smartphones.

A second possible limitation lies in the choice of databases. For our systematic search, we focused on more medical and social sciences–oriented databases. However a preliminary search conducted on a more technology-oriented database (ACM) resulted in a long list of peer-reviewed articles mainly focused on system design, which we would have excluded from the final sample since our research interest was on the impact of mobile technology on health outcomes.

### Conclusion

With this systematic literature review, we sought to map a field that is becoming more and more visible in the literature. This review is an essential first step to the understanding of strengths and limitations in mHealth research.

Some questions remain about the lack of information on the newest technological opportunities and on the best methodologies to assess mHealth’s impact on health outcomes. Is scientific literature the appropriate place to find studies on the effectiveness of mobile applications? Are we exploiting all the potential of smartphones? Why is the scientific research not keeping up with the market evolution? Further research will help to answer these questions.

## Abbreviations

ACM: Association for Computing Machinery
CINAHL: Cumulative Index to Nursing and Allied Health Literature
MMS: multimedia messaging service
SMS: short message service
